# Polymorphisms in Four Genes (*KCNQ1* rs151290, *KLF14* rs972283, *GCKR* rs780094 and *MTNR1B* rs10830963) and Their Correlation with Type 2 Diabetes Mellitus in Han Chinese in Henan Province, China

**DOI:** 10.3390/ijerph13030260

**Published:** 2016-02-26

**Authors:** Kaiping Gao, Jinjin Wang, Linlin Li, Yujia Zhai, Yongcheng Ren, Haifei You, Bingyuan Wang, Xuli Wu, Jianna Li, Zichen Liu, Xiong Li, Yaxin Huang, Xin-Ping Luo, Dongsheng Hu, Kinji Ohno, Chongjian Wang

**Affiliations:** 1 Department of Preventive Medicine, School of Medicine, Shenzhen University, Shenzhen 518060, China; wxl@szu.edu.cn (X.W.); lijianna@szu.edu.cn (J.L.); daisysg@szu.edu.cn (Z.L.); lixiong1@email.szu.edu.cn (X.L.); hyx413459261@sina.com (Y.H.); lxp2005@szu.edu.cn (X.-P.L.); hud@szu.edu.cn (D.H.); 2 Department of Traditional Chinese Medicine Prevention, Preventive Medicine Research Evaluation Center, Henan University of Traditional Chinese Medicine, Zhengzhou 450001, China; wangjinjin510@163.com; 3 Department of Epidemiology, College of Public Health, Zhengzhou University, Zhengzhou 450001, China; lilinlin@zzu.edu.cn (L.L.); zhaiyujiamodi@163.com (Y.Z.); ryc12@sina.com (Y.R.); youhaifei1987@163.com (H.Y.); wangby95@163.com (B.W.); 4 Division of Neurogenetics, Center for Neurological Diseases and Cancer, University Graduate School of Medicine, Nagoya 4668550, Japan; ohnok@med.nagoya-u.ac.jp

**Keywords:** single nucleotide polymorphism, *KCNQ1*, type 2 diabetes, risk factors

## Abstract

Genetic variants at *KCNQ1* rs151290, *KLF14* rs972283, *GCKR* rs780094 and *MTNR1B* rs10830963 have been associated with type 2 diabetes mellitus (T2DM), but the results are contradictory in Chinese populations. The aim of the present study was to investigate the association of these four SNPs with T2DM in a large population of Han Chinese at Henan province, China. Seven-hundred-thirty-six patients with T2DM (cases) and Seven-hundred-sixty-eight healthy glucose-tolerant controls were genotyped for *KCNQ1* rs151290, *KLF14* rs972283, *GCKR* rs780094 and *MTNR1B* rs10830963. The association of genetic variants in these four genes with T2DM was analyzed using multivariate logistic regression. Genotypes and allele distributions of *KCNQ1* rs151290 were significantly different between the cases and controls (*p* < 0.05). The AC and CC genotypes and the combined AC + CC genotype of rs151290 in *KCNQ1* were associated with increases risk of T2DM before (OR = 1.482, 95% CI = 1.062–2.069; *p* = 0.021; OR = 1.544, 95% CI = 1.097–2.172, *p* = 0.013; and OR = 1.509, 95% CI = 1.097–2.077, *p* = 0.011, respectively) and after (OR = 1.539, 95% CI = 1.015–2.332, *p* = 0.042; OR = 1.641, 95% CI = 1.070–2.516, *p* = 0.023; and OR = 1.582, 95% CI = 1.061–2.358, *p* = 0.024; respectively) adjustment for sex, age, anthropometric measurements, biochemical indexes, smoking and alcohol consumption. Consistent with results of genotype analysis, the C allele of rs151290 in *KCNQ1* was also associated with increased risk of T2DM (OR = 1.166, 95% CI = 1.004–1.355, *p* = 0.045). No associations between genetic variants of *KLF14* rs972283, *GCKR* rs780094 or *MTNR1B* rs10830963 and T2DM were detected. The AC and CC genotypes and the C allele of rs151290 in *KCNQ1* may be risk factors for T2DM in Han Chinese in Henan province.

## 1. Introduction

In recent years, the prevalence of diabetes mellitus (DM) has increased significantly in China. In September 2010, a large epidemiological survey [1] found that the prevalence of DM was 9.7% in the Chinese population, suggesting that the overall prevalence of diabetes among nearly 100 million people in China was equivalent to 1/3 of the prevalence worldwide. Among them, 90% of the patients have type 2 diabetes mellitus (T2DM). There are many risk factors associated with developing T2DM, including obesity, unhealthy diet, older age, high blood pressure and family history of diabetes. A genetic background has also been shown to be correlated with T2DM risk. Gene polymorphism studies have revealed that multiple single nucleotide polymorphisms (SNPs) are involved in the development of T2DM. Therefore, genetic risk factors of T2DM need to be explored for T2DM prevention and treatment. Data from the genome-wide association studies (GWAS) [2,3] identified new genes associated with fasting glucose, insulin secretion and sensitivity. These recently identified genes include those in the potassium voltage-gated channel KQT-like subfamily, member 1 (*KCNQ1*) [4], glucokinase regulatory protein (*GCKR*), melatonin receptor 1B (*MTNR1B*) and Krüppel-like factor 14 (*KLF14*) [5]. Most studies suggest that *KCNQ1*rs151290, rs2237892, rs2237895 and rs2237897 polymorphisms are associated with susceptibility to T2DM such as those in Germany [4], China [6], Pakistan and the Netherlands [7,8]. However, the association was not found in Tunisian Arabs or in the Tuebingen Lifestyle Intervention Program in Germany [9,10]. *GCKR* rs780094 and *MTNR1B* rs10830963 were both identified in GWAS in European and Japanese populations [11,12]. The distribution of these two SNPs has also been studied in Chinese populations, but the results are inconsistent [13]. The variant *KLF14* rs972283 may be a risk factor for metabolic disease and had a nominal association with T2DM in a Japanese population, but the association was not significant after Bonferroni’s correction [14]. Although it has been mentioned that it is not risk factor for T2DM in Ningxia province, there may be differences between populations in different regions [15].

Therefore, each of the SNPs above may have different associations with T2DM in different populations. More evidence is currently needed to assess whether they are really a risk for T2DM in Chinese populations. In this study, we investigated these four recently identified SNPs *KCNQ1* rs151290, *KLF14* rs972283, *GCKR* rs780094 and *MTNR1B* rs10830963 in Han Chinese in Henan Province to determine their underlying genetic effects in relation to T2DM in this population.

## 2. Methods

### 2.1. Ethics Statement

The procedure of the study was approved by the Medical Ethics Committee, School of Medicine, Shenzhen University and Zhengzhou University Medical Ethics Committee (No. 2014-2-1310-001; 2014-3-1307-002), and written informed consent was obtained from all participants.

### 2.2. Study Population

The study population included 736 patients with T2DM and 768 healthy controls, randomly selected from an existing large case-control population from Henan Province, China [16]. The patients were recruited from The First Affiliated Hospital of Zhengzhou University and Henan Provincial Armed Police General Hospital. The healthy control population was recruited from the local communities of Xin’an County in Henan Province. All of the participants were from Northern Han Chinese backgrounds. Details of demographic characteristics of the study subjects were collected by interview in person using standard questionnaires [16]. For this study, smoking status was classified as smokers and non-smokers. Participants who currently smoked and/or had smoked at least 100 cigarettes during their lifetime were classified as smokers; Self-reported alcohol consumption was classified as consuming 100 mL liquor in 30 days. The clinical symptoms of diabetes were frequent urination, strong thirst, strong hunger even after having eaten, extreme fatigue, blurred vision, slow healing cuts or bruises tingling, pain, or numbness in the hands/feet. If these symptoms were present alongside fasting blood glucose (FBG) ≥ 7.00 mmol/L or 2 h plasma glucose ≥ 11.0 mmol/L during an oral glucose tolerance test, then T2DM was diagnosed.

Patients were included if they were between 20 and 85 years old, and had been diagnosed with T2DM in accordance with 2005 American Diabetes Association criteria [17]. Participants were excluded if they had low body weight, malnutrition (body mass index (BMI) < 18.5), were diagnosed with type 1 diabetes (fasting insulin < 5 μIU/mL, fasting glucose > 6.8 mmol/L) or other abnormal glucose tolerance, were pregnant, a physical disability, were mentally disturbed, obese (caused by disease), taking certain drugs which could induce obesity (exogenous corticosteroids such as prednisone and long-term oral contraceptives), or had cancer. We consecutively recruited controls from subjects when underwent regular physical examinations at the hospital. Healthy controls were selected on the following criteria: (1) aged between 25 and 75 years; (2) had no history of diabetes; (3) with normal FBG level, no abnormal glucose tolerance; (4) no other chronic diseases; and (5) no malnutrition or low body weight.

The subjects self-reported their ancestry. An interviewer-administered questionnaire was used to collect demographic and anthropometric characteristics.

### 2.3. Clinical and Biochemical Measurements

Body weight, height, waist circumference (WC), and blood pressure (systolic and diastolic: SBP and DBP) were collected as anthropometric data. Blood pressure was measured by an electronic sphygmomanometer. Fasting glucose, low-density lipoprotein cholesterol (LDL-C), high-density lipoprotein cholesterol (HDL-C), plasma total cholesterol (TC), and triglycerides (TG) were collected as laboratory measurements [18]. The “normal” levels of these measurements were defined by the following standards: SBP < 140 mmHg, DBP<90 mmHg, TC 3.35–6.45 mmol/L, TG 0.48–0.88 mmol/L, HDL-C 1.09–1.73 mmol/L and LDL-C 2.7–3.36 mmol/L.

### 2.4. Sample Size Calculation

The sample size for this case-control study was calculated with Sample Size Calculations software (Mark Woodward, The George Institute International Health; Lesley Francis, MIS Consultants Pty. Ltd., New South Wales, Australia).

Minor allele frequency (MAF) of the 4 SNPs (*KCNQ1* rs151290, *KLF14* rs972283, *GCKR* rs780094 and *MTNR1B* rs10830963) were reported to be 0.433, 0.667, 0.366 and 0.634, respectively, shown in Supplementary Table S1. *p*_0_ = 0.366 was set based on the lowest MAF. With OR = 1.5, α = 0.05 (two-tailed), β = 0.10, the estimated sample size was 1058, with 529 for each group.

### 2.5. SNP Selection and Genotyping

A panel of 4 SNPs in 4 diabetes-associated genes were selected on the basis of the following criteria. (1) SNPs in genes that are known to be implicated in fasting glucose, insulin secretion and sensitivity; (2) SNPs previously reported to be associated with T2DM; (3) MAF ≥ 0.366 in Chinese based on the International HapMap Project [19]; (4) Potentially functional SNPs (e.g., coding SNPs and SNPs in Untranslated Regions (UTR), promoter and splicing site).

**Figure 1:**
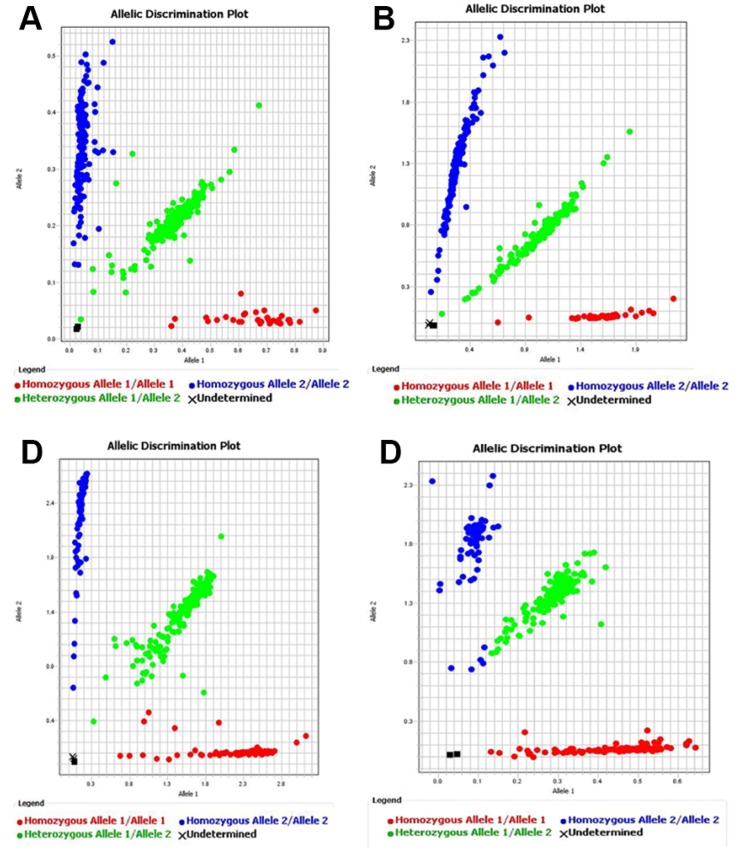
TaqMan single nucleotide polymorphism genotyping assay. (**A**) *KCNQ1*, rs151290, Context Sequence [VIC/FAM] = [A/C]; (**B**) *KLF14*, rs972283, Context Sequence [VIC/FAM] = [A/G]; (**C**) *GCKR*, rs780094, Context Sequence [VIC/FAM] = [C/T] or [A/G]; (**D**) *MTNR1B*, rs10830963, Context Sequence [VIC/FAM] = [C/G].

Genotyping of all SNPs was performed using the TaqMan SNP Genotyping Fluorescence quantitative assays (Applied Biosystems, Foster City, CA, USA) in 2014. The DNA samples were processed in 384-well plates. The TaqMan genotyping reaction was amplified on a GeneAmp PCR system 7000, and fluorescence was detected on an ABI PRISM 7000 sequence detector (Applied Biosystems). The TaqMan Fluorescence SNP probes were synthesized by Life Technologies Biotech Co. (Foster, CA, USA). Overall, genotyping success rate was 100% for rs151290, rs972283, rs780094 and rs10830963, and the error rate was 0% (Figure 1). To verify the reproducibility, we repeated 20% of samples at random as a quality control for genotyping, and the concordance rate was 100% (data not shown). Detailed characteristics of the 4 SNP probes are in Supplementary Table S2, and verification testing data are in Supplementary Table S3.

### 2.6. Statistical Analysis

Statistical analysis was performed using SPSS v17.0 for Windows (SPSS Inc., Chicago, IL, USA). The categorical variables are presented as the number and percentage that they were analyzed with by chi-square test. The continuous variables are presented by median values and the 25th, 75th quartile for non-normally distributed data. The differences between the patients and control populations were assessed by Mann–Whitney–Wilcoxon and Kruskal–Wallis rank tests. The Hardy–Weinberg equilibrium was calculated in controls using chi-square test, and each SNP was separately calculated for cases and controls by use of Fisher’s exact test. Logistic regression analysis was used to calculate the odds ratios (ORs), 95% confidence intervals (95% CIs) and corresponding *p* values for risk of T2DM after adjusting for gender, age, smoking, alcohol consumption, anthropometric measurements including BMI, WC and blood pressure and biochemical indexes including HDL-C, LDL-C, TC and TG levels. Multiplicative interaction terms were included in multivariate logistic regression models to assess the association of interactions between these 4 SNPs with T2DM development, adjusting for gender, age, BMI, smoking and alcohol consumption. The tests were all two-sided and were considered to be statistically significant if *p* < 0.05. The power calculation was performed using PGA software [20].

## 3. Results 

We included 736 T2DM patients (426 males) and 768 controls (324 males). Clinical characteristics of the case and control groups are shown in Table 1 and Supplementary Table S4. Compared with controls, diabetic patients had significantly higher anthropometric and metabolic measurements for BMI, WC, SBP, DBP, and TC, TG, LDL-C and HDL-C levels (*p* < 0.001) as well as age, gender (*p* < 0.001) and alcohol consumption (*p* < 0.006).

The genotype and allele distributions of all four SNPs were in accordance with the Hardy–Weinberg equilibrium proportions in control (*p* > 0.1 or *p* < 0.1 and the sample size was large enough). The genotype and allele distribution of *KCNQ1* rs151290 showed a difference between T2DM patients and healthy controls (*p* < 0.05) (Table 2).

Before and after adjusting for sex, age, anthropometric measurements, biochemical indexes, smoking and alcohol consumption, the AC and CC genotype and the combined AC + CC genotype of *KCNQ1* rs151290 were significantly associated with higher risk of T2DM (OR = 1.482, 95% CI = 1.062–2.069; 1.544, 1.097–2.172; 1.509, 1.097–2.077; adjusted OR = 1.539, 95% CI = 1.015–2.332; 1.641, 1.070–2.516; 1.582, 1.061–2.358, respectively; *p* < 0.05). The C allele of *KCNQ1* rs151290 was also associated with increased risk of T2DM (OR = 1.166, 95% CI = 1.004–1.355, *p* < 0.05). However, the other three SNPs (*KLF14* rs972283, *GCKR* rs780094 and *MTNR1B* rs10830963) were not found to be associated with T2DM (*p* > 0.05) in this Han people in Henan province, China (Table 3).

In the subsequent SNP-SNP interaction analysis, after adjustment for sex, age, BMI, smoking and alcohol consumption, interactions were found between GG genotype of rs10830963 and AG genotype of rs780094 from *MTNR1B* and *GCKR* (OR = 0.392, 95% CI = 0.157–0.982, *p* = 0.046), and CC genotype of rs151290 with AA genotype of rs780094 from *KCNQ1* and *GCKR* (OR = 4.883, 95% CI = 1.366–17.464, *p* = 0.015) in T2DM patients (Table 4).

## 4. Discussion

The aim of this investigation was to analyze the association of *KCNQ1* rs151290, *KLF14* rs972283, *GCKR* rs780094 and *MTNR1B* rs10830963 polymorphisms with T2DM in Han Chinese people in Henan province, China. Our findings reveal an association of genetic variants of the *KCNQ1* rs151290 SNP and increased risk of T2DM in this population. Furthermore, we found that interactions among *KLF14* rs972283, *GCKR* rs780094 and *MTNR1*B rs10830963 were also associated with T2DM.

Variants in *KCNQ1* (the rs151290, rs2237892, rs2237895 and rs2237897 polymorphisms) are suggested to be associated with susceptibility to T2DM through affecting upon incretin and insulin secretion [4]. The rs2237892 CC/TC genotype was related to first- and second-phase insulin secretion in Chinese studies [6]. While the C allele of rs151290 in *KCNQ1* (located within intron 16) has been significantly associated with reduced first-phase glucose-stimulated insulin secretion in different populations [8,21], it may also be associated with lipid metabolism [21]. However, not all studies have found an association between rs151290 and T2DM [9,10]. We found the AC and CC genotype and the combined genotype AC + CC of *KCNQ1* rs151290 was associated with T2DM, and the C allele of *KCNQ1* rs151290 was also associated with T2DM before adjusting for gender, age, anthropometric measurements, biochemical indexes, smoking and alcohol consumption. According to previous study, the risk allele C of rs151290 in *KCNQ1* was associated with increased risk of T2DM in a global population. However, results from studies in several different regions in China, including Hebei, Kunming and Huaihai, were not in agreement with that data from another study in Hebei province [15]. Hebei is a province next to Henan province in China. The similarity of study results between us and that in Hebei may indicate the influence of other risk factors, including lifestyle and genetic background and may affect the association of *KCNQ1* rs151290 and T2DM.

Associations between *KLF14* and T2DM are found in some populations but not in others. In this study, there was no significant association of rs972283 and T2DM, which may be consistent with the result from research by Long *et al.* [22] in African Americans, but is not in accordance with research by Ohshige *et al.* [23] in Japan, Voight *et al.* [2] in Europe and Rees *et al.* [7] in Pakistan. In the Japanese population studied by Ohshige *et al.*, the association of risk allele G of *KLF14* rs972283 and T2DM vanished after being adjusted for sex, age and log-transformed BMI, and there seemed to be no significant association between *KLF14* rs972283 and glucose metabolism [23]. The present study also did not detect the association between *KLF14* rs972283 with T2DM, leaving the genotype as a yet-to-confirm risk factor of the disease.

*GCKR* rs780094 is associated with elevated fasting serum triacylglycerol, and reduced fasting insulin secretion, which reduces the risk of T2DM [24]. The *GCKR* rs780094 A allele may decrease insulin secretion and reduce plasma glucose and TG levels, thereby reducing the risk of T2DM [3,25,26]. However, these results were not confirmed by another study T2DM in a Chinese population [13]. *GCKR* rs780094 polymorphism was shown to be associated with T2DM in Asian populations. In a Japanese population, it was revealed that the rs780094 G allele was closely related to the pathogenesis of T2DM, increased fasting insulin and reduced TG levels, with the A allele associated with low fasting glucose and high TG levels [12]. It has been shown that the T allele of *GCKR* rs780094 is associated with increased blood glucose levels, insulin secretion, TG levels and incidence of T2DM in a German population [11]. However, studies in the Chinese Han population have not reached the same conclusion of an association of rs780094 with T2DM islet β-cell function [27,28]. Two studies investigated the association of *GCKR* rs780094 and T2DM: one in 2009 in Beijing showed that the G allele was a risk factor of T2DM, and the A allele may increase β-cell function [29]; the other study, in 2011 in Shanghai, found no association of the G allele and T2DM, but the authors did not check β-cell function and insulin resistance [27]. Our data showed no direct association with T2DM, but we found that, when interacting with GG genotype of *MTNR1B* rs10830963, the AG genotype of rs780094 may be a protective factor.

*MTNR1B* rs10830963 is significantly associated with decreased β cell function or increased fasting plasma glucose [28,30,31]; studies in Japanese [32] and Mexican Americans [33] have confirmed the similar results. In relation to the decreased β cell function, there seems to be a particular relationship with this polymorphism and the development of gestational diabetes in pregnant women [34], which was also found in a Chinese population [35]. From 2009 to 2010, the association in China was investigated; *MTNR1B* was genotyped in residents of Shanghai: all three studies showed *MTNR1B* rs10830963 was associated with elevated FPG and impaired insulin secretion [28,30,36], similar to a Hong Kong report [31]. Hu *et al.* reported that the *MTNR1B* genetic variantswere associated with first-phase but not second-phase insulin secretion [36]. However, Liu *et al.* reported that *MTNR1B* rs10830963 was associated with fasting glucose and β-cell function in the Shanghai but not in a Beijing subpopulation [30]. Therefore, *MTNR1B* rs10830963 research in China has not produced consistent results. Our data complements and increases the population information for different regions in China, as we did not detect an association with T2DM. This suggests that the CG genotype of *MTNR1B* rs10830963 is not associated with T2DM in Han Chinese in the Henan province, similar to results from residents of Beijing [30]. However, the lack of an association might be affected by the sample size of the study and other confounding factors, so more detailed analysis is required to support this finding.

The suggestion that differences in the associations of these polymorphisms with T2DM may vary with different populations within China may seem surprising. However, a study of more than 6000 Han Chinese from 10 provinces in China showed differences between northern and southern residents, with geographic disparities in DNA [37]. In addition, China is divided into north and south approximately by the Yangtze River, and the environment, diet structures and lifestyles differ greatly, which might also explain the inconsistent results. Therefore, it is not strange that differences occur between North and South China, such as Beijing, Shanghai and Hong Kong, with the association of SNPs (*GCKR* rs780094 and *MTNR1B* rs10830963) with T2DM, as has been reported [15,28,29,30].

## 5. Conclusions

In conclusion, our findings suggest that genotype and allele distributions of *KCNQ1* rs151290 are different between T2DM patients and controls, and the variant genotypes and allele *KCNQ1* rs151290 are associated with increased risk of T2DM in Han Chinese people in Henan province, China. In addition, there may be interactions between AG, GG and CG genotypes of *KLF14* rs972283, *GCKR* rs780094 and *MTNR1B* rs10830963.

## Figures and Tables

**Table 1:** Characteristics of type 2 diabetes mellitus (T2DM) cases and controls.

Characteristics	Cases (*n* = 736)	Controls (*n* = 768)	Z/χ^2^	*p*
Gender *				
Male	426 (57.88)	324 (42.19)	37.018	<0.001
Female	310 (42.12)	444 (57.81)		
Age ^#^	52.50 (43–61)	47.00 (39–57)	6.212	<0.001
FBG ^#^	7.04 (5.73, 9.21)	5.19 (4.92, 5.50)	24.350	<0.001
BMI ^#^ (kg/m^2^)	28.58 (25.37, 32.12)	23.50 (21.43, 25.85)	20.671	<0.001
WC ^#^ (cm)	93.50 (85.00, 108.00)	80.50 (74.53, 87.70)	20.442	<0.001
SBP ^#^ (mmHg)	128.00 (120.00, 138.00)	121.50 (111.33, 134.67)	7.011	<0.001
DBP ^#^ (mmHg)	82.00 (72.00, 90.00)	77.00 (71.33, 85.33)	6.147	<0.001
TC ^#^ (mmol/L)	4.87 (4.29, 5.59)	4.30 (3.70, 4.91)	12.398	<0.001
TG ^#^ (mmol/L)	1.59 (1.11, 2.37)	1.30 (0.95, 1.88)	6.652	<0.001
HDL-C ^#^ (mmol/L)	1.09 (0.96, 1.25)	1.15 (0.99, 1.32)	−3.378	<0.001
LDL-C ^#^ (mmol/L)	3.03 (2.55, 3.69)	2.40 (1.93, 2.90)	16.713	<0.001
Smoking *				
Non-smoking	536 (48.73)	565 (51.27)	0.124	0.725
Smoking	20 (49.75)	203 (50.25)		
alcohol consumption *				
Non-drinking	611 (47.36)	679 (52.64)	7.601	0.0058
Drinking	121(57.62)	89(42.38)		

* *n* (%), chi-square test; ^#^ median (25th and 75th quartile), Mann–Whitney–Wilcoxon test. FBG: fasting blood glucose; BMI: body mass index; WC: waist circumference; SBP: systolic blood pressure; DBP: diastolic blood pressure; TC: total cholesterol; TG: Triglyceride; HDL-C: high-density lipoprotein cholesterol; LDL-C: low-density lipoprotein cholesterol.

**Table 2:** Distribution of genotype and allele frequncy of 5 single nucleotide polymorphisms (SNPs) in T2DM cases and controls.

SNP	Genotype	*n* for Genotype		*p* (G) *	Allele	*n* for Allele		*p* (A) *	*p* (HW) ^#^
		Case	Control			Case	Control		
rs151290 (KCNQ1)	AA/AC/CC	71/358/294	107/364/287	0.037	A/C	500/946	578/938	0.045	0.6237
rs972283 (KLF14)	GG/AG/AA	379/286/56	389/297/71	0.540	G/A	1044/398	1075/439	0.400	0.1942
rs780094 (GCKR)	GG/AG/AA	194/343/185	186/348/225	0.204	G/A	731/713	720/798	0.082	0.0264
rs10830963 (MTNR1B)	CC/CG/GG	243/347/134	280/350/129	0.387	C/G	833/615	910/608	0.181	0.2737

* Fisher’s exact test; ^#^ chi-square test; *p* (G): *p* for genotype; *p* (A): *p* for allele; *p* (HW): *p* for Hardy-Weinberg equilibriumte in control.

**Table 3:** Association of genetic variants with type 2 diabetes.

SNP	Genetic Model	Cases/Controls	OR (95% CI)	*p*	Adjusted OR (95% CI) *	Adjusted *p* *
rs151290	Genotype					
(*KCNQ1*)	AA	71/107	1.000		1.000	
	AC	358/364	1.482 (1.062–2.069)	0.021	1.539 (1.015–2.332)	0.042
	CC	294/287	1.544 (1.097–2.172)	0.013	1.641 (1.070–2.516)	0.023
	Dominant model					
	AA	71/107	1.000			
	AC + CC	652/651	1.509 (1.097–2.077)	0.011	1.582 (1.061–2.358)	0.024
	Recessive model					
	AA + AC	429/471	1.000		1.000	
	CC	294/287	1.125 (0.913–1.386)	0.270	1.154 (0.893–1.491)	0.275
	Allele					
	A	500/578	1.000			
	C	946/938	1.166 (1.004–1.355)	0.045		
rs972283	Genotype					
(*KLF14*)	GG	379/389	1.000		1.000	
	AG	286/297	0.988 (0.797–1.226)	0.915	0.901 (0.692–1.173)	0.438
	AA	56/71	0.810 (0.555–1.181)	0.273	0.734 (0.458–1.176)	0.199
	Dominant model					
	GG	379/389	1.000		1.000	
	AG + AA	342/368	0.954 (0.778–1.170)	0.650	0.870 (0.677–1.118)	0.275
	Recessive model					
	GG + AG	665/686	1.000		1.000	
	AA	56/71	0.814 (0.564–1.173)	0.270	0.768 (0.486–1.213)	0.258
	Allele					
	G	1044/1075	1.000			
	A	398/439	0.934 (0.795–1.096)	0.400		
rs780094	Genotype					
(*GCKR*)	GG	194/186	1.000		1.000	
	AG	343/348	0.945 (0.736–1.214)	0.658	1.090 (0.800–1.485)	0.585
	AA	185/225	0.788 (0.596–1.043)	0.096	0.863 (0.610–1.221)	0.404
	Dominant model					
	GG	194/186	1.000		1.000	
	AG + AA	528/573	0.883 (0.700–1.116)	0.298	1.001 (0.749–1.338)	0.994
	Recessive model					
	GG + AG	537/534	1.000		1.000	
	AA	185/225	0.818 (0.651–1.027)	0.084	0.815 (0.615–1.079)	0.153
	Allele					
	G	731/720	1.000			
	A	713/798	0.880 (0.762–1.017)	0.082		
rs10830963	Genotype					
(*MTNR1B*)	CC	243/280	1.000		1.000	
	CG	347/350	1.142 (0.910–1.433)	0.251	1.026 (0.775–1.357)	0.858
	GG	134/129	1.197 (0.890–1.610)	0.235	1.128 (0.788–1.615)	0.510
	Dominant model					
	CC	243/280	1.000		1.000	
	CG + GG	481/479	1.157 (0.935–1.432)	0.181	1.054 (0.811–1.370)	0.694
	Recessive model					
	CC + CG	590/630	1.000		1.000	
	GG	134/129	1.109 (0.850–1.448)	0.446	1.112 (0.807–1.532)	0.517
	Allele					
	C	833/910	1.000			
	G	615/608	1.105 (0.955–1.279)	0.181		

* Adjusted for sex, age, anthropometric measurements, biochemical indexes, smoking and alcohol consumption; OR, odds ratio; 95% CI, 95% confidence interval.

**Table 4:** The impact of interaction among SNPs on the risk of type 2 diabetes mellitus.

SNP *	Genotype	SNP *								
		rs10830963 (*MTNR1B*)			rs151290 (*KCNQ1*)			rs972283 *(KLF14*)		
		CC	CG	GG	AA	AC	CC	GG	AG	AA
rs780094 (*GCKR*)	GG	1 (Reference)	1.113 (0.389–3.180) 0.842	2.347 (0.626–8.791) 0.206	1 (Reference)	0.567 (0.193–1.671) 0.304	0.466 (0.157–1.382) 0.169	1 (Reference)	1.044 (0.379–2.878) 0.933	1.822 (0.085–38.920) 0.701
	AG	1.170 (0.401–3.420) 0.774	0.655 (0.313–1.368) 0.260	**0.392 (0.157**–**0.982) 0.046**	1.170 (0.401–3.420) 0.774	0.976 (0.331–2.875) 0.965	1.153 (0.383–3.472) 0.800	1.170 (0.401–3.420) 0.774	1.004 (0.513–1.965) 0.991	3.393 (0.955–12.055) 0.059
	AA	0.396 (0.115–1.362) 0.142	1.304 (0.569–2.986) 0.531	0.442 (0.156–1.249) 0.124	0.396 (0.115–1.362) 0.142	2.265 (0.657–7.812) 0.196	**4.883 (1.366**–**17.464) 0.015**	0.396 (0.115–1.362) 0.142	1.245 (0.583–2.659) 0.571	2.583 (0.672–9.934) 0.167
rs151290 (*KCNQ1*)	AA	1 (Reference)	1.113 (0.389–3.180) 0.842	2.347 (0.626–8.791) 0.206	-	-	-	-	-	-
-	AC	0.567 (0.193–1.671) 0.304	1.046 (0.400–2.738) 0.927	0.721 (0.217–2.396) 0.594	-	-	-	-	-	-
-	CC	0.466 (0.157–1.382) 0.169	0.905 (0.336–2.438) 0.844	0.521 (0.151–1.797) 0.302	-	-	-	-	-	-
rs972283 *(KLF14*)	GG	1 (Reference)	1.113 (0.389–3.180) 0.842	2.347 (0.626–8.791) 0.206	1 (Reference)	0.567 (0.193–1.671) 0.304	0.466 (0.157–1.382) 0.169	-	-	-
	AG	1.044 (0.379–2.878) 0.933	0.801 (0.437–1.467) 0.472	1.133 (0.512–2.508) 0.757	1.044 (0.379–2.878) 0.933	1.034 (0.426–2.510) 0.942	1.128 (0.452–2.814) 0.796	-	-	-
	AA	1.822 (0.085–38.920) 0.701	1.940 (0.627–6.002) 0.250	1.986 (0.482–8.187) 0.343	1.822 (0.085–38.920) 0.701	0.204 (0.011–3.892) 0.291	0.222 (0.011–4.495) 0.327	-	-	-

Data are OR (95% CI), *p* value; * data are adjusted for sex, age, body mass index, smoking and alcohol consumption; *p* value for testing effect of modification by behavior risk factors using an interaction term of status of b.

## Supplementary Materials

The following are available online at www.mdpi.com/1660-4601/13/3/260/s1. Table S1: Single nucleotide polymorphisms (SNPs) selected for study in Han Chinese in Henan province, China. Table S2: Single nucleotide polymorphism (SNP) probes used for genotyping. Table S3: Verification primers. Table S4: Clinical characteristics of T2DM cases and controls in terms of biochemical measurements (SBP, DBP, TC, TG, HDL-C, LDL-C).
