# Supplementary material for: Polymorphisms in Four Genes (KCNQ1 rs151290, KLF14 rs972283, GCKR rs780094 and MTNR1B rs10830963) and Their Correlation with Type 2 Diabetes Mellitus in Han Chinese in Henan Province, China
Source: Int J Environ Res Public Health. 2016 Feb 26;13(3):260. doi: 10.3390/ijerph13030260 (PMC4808923; doi:10.3390/ijerph13030260)
Supplement: Supplementary file 1 [file ijerph-13-00260-s001.pdf]

Supplementary Materials: Polymorphisms in Four Genes (*KCNQ1* rs151290, *KLF14* rs972283, *GCKR* rs780094 and *MTNR1B* rs10830963) and Their Correlation with Type 2 Diabetes Mellitus in Han Chinese in Henan Province, China

Kaiping Gao, Jinjin Wang, Linlin Li, Yujia Zhai, Yongcheng Ren, Haifei You, Bingyuan Wang, Xuli Wu, Jianna Li, Zichen Liu, Xiong Li, Yaxin Huang, Xin-Ping Luo, Dongsheng Hu, Kinji Ohno and Chongjian Wang

Table S1. Single nucleotide polymorphisms (SNPs) selected for study in Han Chinese in Henan province, China.

Gene	SNP	Allele	Ancestral Allele	MAF in CHB
KCNQ1	rs151290	A/C	A	0.433
KLF14	rs972283	G/A	G	0.667
GCKR	rs780094	G/A	G	0.366
MTNR1B	rs10830963	C/G	C	0.634

MAF, minor allele frequency; CHB, Han Chinese in Beijing.

Table S2. Single nucleotide polymorphism (SNP) probes used for genotyping.

Gene Name	SNP	TaqMan Probe Context Sequence
KCNQ1	rs151290	CCTCAGTGCAGGCAGCTGAGCCCAG[A/C]CCCCTGGG CTGTCCTTGGAACCACT
KLF14	rs972283	TGTATACTTGGAAAAAATCCTACCT[A/G]GTCATGGC GCATACTTATTTTTACA
GCKR	rs780094	CTCAACAAATGTATTGATCAGCAAA[C/T]ATGTGTCA GTCATGGTCTAAAAAA
MTNR1B	rs10830963	GTGATGCTAAGAATTCACACCATCT[C/G]CTATCCAG AACCAGTAACTGCCTGG

Table S3. Verification primers.

Name	GKP Primer Sequence	Product Length (bp)
KCNQ1-rs151290-F	ACTTCCTGCTCCATCCCAGT	297
KCNQ1-rs151290-R	CTCAGAGCAGGCAAAGTGG	
KLF14-rs972283-F	CTCCTCCCCATTTCTCATCA	297
KLF14-rs972283-R	CCAAGAAAATACAAAGAGGAAAGG	
GCKR-rs780094-F	CATGTTCTCTGAGTCCTTCCA	296
GCKR-rs780094-R	AGGCTTGTTGAGAACTCCTGA	
MTNR1B-rs10830963-F	GAATTGGCATTTCTGGGGTA	397
MTNR1B-rs10830963-R	CTAGGCCTTCCAGAGCCTTT	

Table S4. Clinical characteristics of T2DM cases and controls in terms of biochemical measurements (SBP, DBP, TC, TG, HDL-C, LDL-C).

**Characteristics**		**Cases (*n* = 738) *n* (%)**	**Controls (*n* = 768) *n* (%)**	***p*** #
SBP * (mmHg)	Normal	557 (75.5)	636 (82.8)	<0.001 #
	Abnormal	181 (24.5)	132 (17.2)	
DBP * (mmHg)	Normal	481 (65.2)	643 (83.7)	<0.001 #
	Abnormal	257 (34.8)	125 (16.3)	
TC * (mmol/L)	Normal	673 (91.2)	760 (99.0)	<0.001 #
	Abnormal	65 (8.8)	8 (1.0)	
TG * (mmol/L)	Normal	101 (13.7)	152 (19.8)	<0.002 #
	Abnormal	637 (86.3)	616 (80.2)	
HDL-C * (mmol/L)	Normal	370 (50.1)	455 (59.2)	<0.001 #
	Abnormal	368 (49.9)	313 (40.8)	
LDL-C * (mmol/L)	Normal	481 (65.2)	698 (90.9)	<0.001 #
	Abnormal	257 (34.8)	70 (9.1)	

*: Chi-square test; #: *p* value between cases and controls were less than 0.002; SBP: systolic blood pressure; DBP: diastolic blood pressure; TC: total cholesterol; TG: Triglyceride; HDL-C: high-density lipoprotein cholesterol; LDL-C: low-density lipoprotein cholesterol; Normal range of the clinical properties has been described in "Biochemical Measurements".

© 2016 by the authors; licensee MDPI, Basel, Switzerland. This article is an open access article distributed under the terms and conditions of the Creative Commons by Attribution (CC-BY) license (http://creativecommons.org/licenses/by/4.0/).
